# Protocol Development for HMU! (HIV Prevention for Methamphetamine Users), a Study of Peer Navigation and Text Messaging to Promote Pre-Exposure Prophylaxis Adherence and Persistence Among People Who Use Methamphetamine: Qualitative Focus Group and Interview Study

**DOI:** 10.2196/18118

**Published:** 2020-09-14

**Authors:** Vanessa M McMahan, Noah Frank, Smitty Buckler, Lauren R Violette, Jared M Baeten, Caleb J Banta-Green, Ruanne V Barnabas, Jane Simoni, Joanne D Stekler

**Affiliations:** 1 San Francisco Department of Public Health Center on Substance Use and Health San Francisco, CA United States; 2 Department of Health Services University of Washington Seattle, WA United States; 3 Department of Global Health University of Washington Seattle, WA United States; 4 Department of Epidemiology University of Washington Seattle, WA United States; 5 Alcohol & Drug Abuse Institute University of Washington Seattle, WA United States; 6 Department of Psychology University of Washington Seattle, WA United States

**Keywords:** pre-exposure prophylaxis, methamphetamine, text messaging, peer navigation, men who have sex with men, transgender people

## Abstract

**Background:**

Cisgender men who have sex with men (MSM) and transgender people (TGP) who use methamphetamine are disproportionately impacted by HIV acquisition. Pre-exposure prophylaxis (PrEP) is highly effective at preventing HIV, and interventions that support PrEP persistence and adherence should be evaluated among MSM and TGP who use methamphetamine.

**Objective:**

We conducted formative work to inform the development of text messaging and peer navigation interventions to support PrEP persistence and adherence among MSM and TGP who use methamphetamine. In this paper, we describe how the findings from these focus groups and interviews were used to refine the study interventions and protocol for the Hit Me Up! study (HMU!; HIV Prevention in Methamphetamine Users).

**Methods:**

Between October 2017 and March 2018, we conducted two focus groups and three in-depth interviews with MSM and TGP who use methamphetamine or who have worked with people who use methamphetamine. During these formative activities, we asked participants about their opinions on the proposed interventions, education and recruitment materials, and study design. We focused on how we could develop peer navigation and text messaging interventions that would be culturally appropriate and acceptable to MSM and TGP who use methamphetamine. Transcripts were reviewed by two authors who performed a retrospective content analysis to describe which specific opinions and recommendations influenced protocol development and the refinement of the interventions.

**Results:**

Overall, participants thought that MSM and TGP would be interested in participating in the study, although they expected recruitment and retention to be challenging. Participants thought that the peer navigator should be someone who is nonjudgmental, has experience with people who use methamphetamine, and is patient and flexible. There was consensus that three text messages per day were appropriate, adherence reminders should be straightforward, all messages should be nonjudgmental, and participants should be able to tailor the timing and content of the text messages. These suggestions were incorporated into the study interventions via the hiring and training process and into the development of the text library, platform selection, and customizability of messages.

**Conclusions:**

It is important to include the opinions and insights of populations most impacted by HIV to develop PrEP interventions with the greatest chance of success. Our formative work generated several recommendations that were incorporated into the interventions and protocol development for our ongoing study.

**Trial Registration:**

ClinicalTrials.gov NCT03584282; https://clinicaltrials.gov/ct2/show/NCT03584282

## Introduction

HIV pre-exposure prophylaxis (PrEP) is a safe and effective method for reducing HIV acquisition that was approved for use in the United States in July 2012 [1-3]. Despite increasing knowledge about and use of PrEP nationally [4], HIV continues to disproportionately impact cisgender men who have sex with men (MSM) and transgender people (TGP) [5,6]. Furthermore, MSM and TGP who use methamphetamine are at particularly high risk for HIV [7-10]. In King County, Washington, USA, HIV incidence among MSM who use methamphetamine is nearly six times greater than that of MSM who do not use methamphetamine [11]. While there is a paucity of data regarding substance use and HIV risk among TGP, there is evidence that methamphetamine use and HIV acquisition disproportionately impact transgender women. Two studies of transgender women in different west coast cities in the United States both found that approximately 20% reported recent methamphetamine use [7,8], and HIV prevalence has been estimated to be 28% among transgender women [12]. While TGP are often excluded from research that focuses on MSM, it is important to include TGP in prevention research in order to address health disparities and target HIV prevention interventions to those at highest risk.

During our preliminary work in Seattle, Washington, we found that HIV-negative MSM and TGP who use methamphetamine had high levels of PrEP knowledge and insurance coverage, but few had enrolled in local PrEP programs [13]. Among people using PrEP, methamphetamine has been shown to be associated with poor adherence [14,15]. In our formative work, we identified both traditional barriers to PrEP uptake, adherence, and persistence (eg, lack of awareness and forgetting doses) and barriers specific to methamphetamine use. Participants suggested that peer navigation and text messaging could be helpful to support MSM and TGP who use methamphetamine to overcome barriers to effective PrEP use [16].

Peer navigation interventions have been used to improve healthy behavior in a wide variety of contexts. For people living with HIV, matching patients with a peer who shares key characteristics or experiences has been shown to improve HIV knowledge and antiretroviral treatment attitudes and decrease substance use [17-20]. Peer navigation may be an effective strategy to support PrEP persistence and adherence as well. Text message interventions also have promise to promote PrEP adherence. A systematic review of text messaging interventions for HIV and sexually transmitted infection (STI) prevention and treatment showed that some interventions were associated with increased HIV testing and self-reported adherence [21]. In one study, participants who received bidirectional text messages were more than twice as likely to be adherent to PrEP compared to those who did not receive text messages. Moreover, the majority of participants (88%) thought that receiving the texts was very or somewhat helpful [22].

In 2017, we received funding from the National Institute on Drug Abuse for the HMU! study (Hit Me Up!; HIV Prevention in Methamphetamine Users) (NCT03584282) to develop and conduct a preliminary evaluation of two interventions—peer navigation and text messaging—designed to promote PrEP adherence and persistence among MSM and TGP who use methamphetamine. This manuscript describes formative work that informed the development and refinement of these interventions and our study protocol.

## Methods

### Procedures

Between October 2017 and March 2018, we conducted two focus groups and three in-depth interviews with MSM and TGP who use methamphetamine or who have worked with people who use methamphetamine (eg, peer educators). Focus groups were facilitated by two authors (VMM and JDS) and interviews were conducted by VMM. Focus groups and in-depth interviews followed semistructured interview guides (see Multimedia Appendices 1 and 2) that included questions regarding what information about PrEP and the study should be provided to participants, what types of recruitment materials should be used, and whether participants thought people would be interested in participating in the study as well as specific questions to develop the peer navigation and text messaging interventions. The questions about the text messaging intervention included reviewing messages used in an earlier study by Reback et al [23], which were aimed at reducing methamphetamine use and sexual behaviors associated with risk for HIV acquisition. These text messages were developed using the Social Support Theory, the Health Belief Model, and the Social Cognitive Theory. The Social Support Theory posits that instrumental, informational, and emotional support from one’s network can lead to positive changes in health behavior. The Health Belief Model describes how one’s beliefs about health behaviors and risks to one’s health are associated with engagement in protective behaviors. Finally, the Social Cognitive Theory describes how the interaction of the individual, their behavior, and the environment are related to health behavior. The study done by Reback et al demonstrated a reduction in methamphetamine use and unprotected anal intercourse with nonprimary partners among MSM who used methamphetamine [24]. Focus groups and interviews were anonymous and the audio recordings were transcribed.

The first focus group was done with 9 peer educators at Project Needle and Sex Education Outreach Network (Project NEON) of the Seattle Counseling Service. Project NEON is a harm reduction program that aims to reduce sexual and drug-related risks associated with the use of methamphetamine. In order to generate preliminary data for this project, the authors had collaborated with the Project NEON peer educators on earlier work to try to better understand PrEP use among MSM and TGP who use methamphetamine and to increase PrEP education in this community [13,16]. This work included developing PrEP education materials with the Project NEON peer educators and recommendations for evaluating text messages and peer navigation as potential supports for PrEP adherence among MSM and TGP who use methamphetamine.

The second focus group was recruited through Seattle Area Support Groups (SASG), now Peer Seattle, a nonprofit organization that provides peer emotional support and development services to lesbian, gay, bisexual, transgender, and queer or questioning individuals, plus other sexual and gender minorities (LGBTQ+). The second group consisted of 10 MSM who had used methamphetamine. For these two focus groups, the researchers gained access to participants through long-standing relationships with these two community organizations.

After conducting these two focus groups, we chose to conduct research activities with individuals who represented perspectives that had not already been shared, specifically LGBTQ+ youth and people who are not cisgender. We originally planned to conduct an additional two focus groups with up to 10 participants each representing these populations; however, we were advised that interviews would be more appealing to individuals who are not cisgender since there was less chance of being “outed” in a one-on-one activity. We recruited eligible interview participants through palm cards and word-of-mouth. Palm cards were brought to local agencies that provide services to LGBTQ+ youth and people who are not cisgender. The researchers also informally provided palm cards to, or discussed the study with, community members in these networks with whom they had up to two decades of collaboration. Despite these various approaches to recruitment, we only conducted three interviews out of the 20 planned.

At the end of each focus group or in-depth interview, participants were given a US $40 gift card to an online retailer. The University of Washington Institutional Review Board approved this study (#00004760). All participants were given an information sheet for participation; written consent was waived.

### Protocol Development

The protocol was drafted in parallel with formative activities. Therefore, after each focus group and interview, the facilitators (VMM and JDS) met to discuss the major themes that arose during the discussion to inform ongoing study development in an iterative process. The first version of the study protocol was finalized in April 2018 and submitted for ethics review and approval.

### Analysis

After completion of the formative work, two authors (VMM and NF) performed a retrospective content analysis [25] of the focus group and in-depth interview transcripts to describe which specific opinions and recommendations influenced protocol development and the study interventions. First, they reviewed transcripts individually and compared them to the final study protocol. Then they met and discussed which themes from the early formative work had most influenced the resulting interventions and protocol until they reached consensus.

## Results

### Overview

Across focus groups and interviews, participants reported that they thought MSM and TGP who use methamphetamine would be interested in the study. Participants offered a variety of suggestions about the peer navigation and text messaging interventions. Their recommendations focused on ways to make these interventions relevant and accessible to MSM and TGP who use methamphetamine.

### Peer Navigation Intervention

Focus group and interview participants thought that the peer should be someone who is patient, nonjudgmental, not transphobic, and has experience with the target population.

I don't want a peer navigator that does not have any comfort with the addiction of methamphetamine. To just assign someone, a peer navigator, that doesn't have a clue about—or hasn't gone through any sort of addiction training—is a disservice to the addict that's actively using.

Interviewee #2

I'm not sure if everyone's comfort level or knowledge level would be the same with a trans man or trans woman. There's a lot of transphobia in the gay community...[So we should make] sure that we have [peers] that are willing...to work with that population.

Interviewee #2

Participants thought that it would be important for the peer to help with appointment reminders and medication support, including refills. One peer recommended the option of holding on to medications for participants, as the peer had prior experience providing a few days’ supply of medications to someone he had sponsored and reported that it had helped with medication compliance. They also recommended that the peer be flexible in order to meet study participants outside of the “9-to-5” schedule and in a variety of settings.

A peer probably would have helped if [they could have] taken the [medication] bottles back for me...[I] probably would have kept going [to the doctor].

Focus group #2, attendee #10

They also discussed barriers the peer should anticipate, including difficulties reaching study participants.

Your peer is going to have trouble reaching people during three-day periods when they’re at the bathhouse.

Focus group #2, attendee #8

Participants discussed how setting boundaries would be important for the peer, including appropriate times to expect them to answer the phone and what interactions would or would not be appropriate if the peer and participant were visible to each other on the internet (eg, on a hookup app).

[MSM and TGP who use methamphetamine] might listen to [a peer who]...can talk about losing teeth and having to go to the dentist or slamming dope or snorting crystal meth or going to orgies and stuff and going to random sex hookups in the bathhouse. [Who] can really kinda get down and talk about that stuff with a complete comfort level but be able to maintain those boundaries that are really important...

Interviewee #2

### Text Messaging Intervention

The number of texts per day that participants thought would be appropriate ranged from two to six and there was general consensus that three per day was not excessive. Focus group and interview participants suggested that the study participants should be allowed to select the timing of the messages, since people who use methamphetamine may not be accessible during the daytime. There were variable responses to the example text messages that were shown to participants (see Table 1).

Some participants liked the content, as evidenced by the following quotes.

I liked [the text message examples]. I feel like they speak to the idea of remaining on your PrEP regimen and how important that is for overall health. I also like the idea that the research is not judging whether or not you’re using meth. It’s really about promoting the idea that we can use drugs if we choose to and still take PrEP.

Interviewee #2

I think [the example text messages] are real. It’s like if your friend sent it to you.

Interviewee #3

The text messages from the Social Support Category were particularly liked.

I just don’t know if I’d listen to any of these things because when I am on meth, I want what I want and I’m very selfish and I don’t care about my family or what they think...But I do like the emotional support, like just having someone there caring. Even if I don’t listen, I am going to remember when I am sober.

Focus group #2, attendee #1

When I’m laying down and going to sleep, I will say, “Thank god someone fucking told me to go to fucking sleep.”

Focus group #2, attendee #2

In addition, text messages that provided “straightforward” information, like “Needle exchange 2nite @ __________,” were preferred, although one participant said receiving these may make them feel guilty.

[The messages that just contain information about services] might cause guilt...My first reaction is “I’ve got to go get an HIV test now that I’m high.”

Focus group #2, attendee #8

Some other examples of potential text messages were regarded less highly.

The ones that are involved with helping me stay adherent and abide by my health stuff, that’s great. But then some of the random—like public health announcement ones—like...“gums bleeding—gargle with peroxide.” That’s just some random health tip. That would get on my nerves. I would be annoyed.

Focus group #2, attendee #4

**Table 1:** Example text messages shared for feedback during formative work.

Theory or model	Example text messages
Social Support Theory	There's no such thing as a good sore. Mix Viagra and Poppers? U may come and then go. Gums bleeding? Gargle with peroxide. Rehydrate and rest!
Health Belief Model	Stop B4 U rub it raw. RU drippin' while UR trippin? Don't let NE1 tell U what UR limits R! Don't let Tina take you down.
Social Cognitive Theory	Sharon Needles? She's bad news. Peace of mind is priceless. Know UR health info, B informed! Needle exchange 2nite @ ______________.

The variety of opinions on our example text messages resulted in us developing many text message options: 540 text messages across 11 content categories.

Focus group and interview participants consistently recommended that study participants should be able to choose their own text message content.

At the point someone [is] signed up to be a part of this [study], maybe [let them select] different categories. “Hey, do you want information or to receive text messages about this subject, this subject, this subject, or this subject?” Because if I don’t want to know about, say, club use or whatever, then I wouldn’t click on that.

Focus group #1, attendee #4

Across focus groups and interviews, there was consensus that straightforward PrEP reminders would be best (eg, “take your pill”) and that we should “change up” the messages so they were not the same each day. Daily PrEP reminders were discussed in the context of the effects of methamphetamine, including “losing days.” One participant recommended including the passage of days within the texts themselves to help participants be aware of what day it is and how much time is passing.

Why not say, “It’s Monday, take your pill. It’s Tuesday, take your pill. It’s Wednesday, take your pill...” It’s letting them know that time has passed.

Focus group #2, attendee #1

Multiple participants recommended using email in conjunction with text messages, because it is common to lose one’s phone.

Email [would be the best way for the peer to help me make appointments], because I have lost my phone so many times. So, yeah, email and text.

Interviewee #1

Participants also emphasized that using emojis was very important and could help “with discretion.”

If you can have [emojis in the text messages], then do it...People are gonna read it, it’s just more appealing, catchy, cute.

Interviewee #3

I think it's okay to send [a pill emoji] and a green heart or a thumbs up or a hug, like a PrEP hug...Because I think it's simple. And if someone's looking over your shoulder, “Well, what do they mean, ‘Take your PrEP?’”...And they can be like, “It's someone saying, ‘Do I have pills, do I have OxyContin’” or whatever. They can play it off however they want. I think it's really important.

Interviewee #2

Finally, in our second focus group, one participant reported a technology phobia when high on methamphetamine and advised that receiving texts could cause paranoia for some.

If I saw [an email and text message reminder to take my PrEP] it would send me into a psychotic little scare. That technology and stuff isn’t awesome at that point. So if I saw something like that pop up, I’d freak out...I might not be thinking of what that was or I might start getting paranoid about it if I’m at that point...

Focus group #2, attendee #10

Endorsement of potential paranoia in this population by focus group attendees led to a protocol exclusion criterion regarding discomfort with technology.

### PrEP Education, Recruitment, and Study Operations

In the focus groups and interviews, we shared educational materials that had been previously developed during formative work done with Project NEON to increase PrEP education and uptake among MSM and TGP who use methamphetamine [19]; we then asked participants for feedback about the card images and content. Participants liked the nonjudgmental phrasing “we aren’t here to judge your drug use” but recommended replacing “your drug use” with “you.” Some, but not all, participants thought that the inclusion of the word “tweaker” was stigmatizing; however, there was consensus that the word “partying” would be a preferable word choice and widely understood among the LGBTQ+ community to refer to methamphetamine use. Using terms familiar to individuals who engage in party and play (ie, having sex while using methamphetamine) was liked and recommended (eg, “parTy” or “PnP” [party and play]). Participants recommended images that did not blatantly show drug use or sex but conveyed these activities discretely (eg, smoke in the background and an image suggestive of a sex act). Images that were gay friendly and transgender inclusive were also endorsed.

[I think a good image could be] a man that's holding a pipe, but it's the picture of the man doing something, and the pipe's just in his hand...

Interviewee #3

What about...getting an image where it doesn't show anything; it’s soft core, but it's suggesting a sex act?

Focus group #1, attendee #3

Participants were concerned that our recruitment approach and materials would not reach a subset of MSM and TGP who use methamphetamine and who may be at particularly high risk for HIV, including those who are homeless and engage in survival sex work.

[The party or die crowd are] isolated. They’re not in bathhouses. They’re not in this scene. They’re not in the gay bars. They’re not seen on Capitol Hill. They’re the unseen ones that are homeless and basically being passed around a crowd of men and drug dealing assholes.

Focus group #2, attendee #5

Participants recommended that we have later hours for study visits (ie, after 5 PM) to make the study more accessible to potential participants.

You’re dealing with an after-5, up-all-night crowd...When are you gonna have those hours that are not in the realm of 9 to 5? Because addicts don’t live a normal kind of day. Their days are kind of upside down, much like the homeless community. We can’t always get them into services at 5 because they don’t have any idea of time...I think that’s gonna be your biggest hurdle—getting people to actually show up during the day when it’s light out.

Interviewee #2

Participants thought that retention may be challenging among MSM and TGP who use methamphetamine and, to retain participants, it would be important to be nonjudgmental.

Ten [per intervention group] is not a lot of people...if three of them start using meth regularly and don’t show up, what happens to your data?...I think [retention is] more of a concern [among MSM and TGP who use methamphetamine than others]...the only thing I think that might help people stay engaged is any lack of judgement. Yeah, any lack of judgement. Like there’s no disapproval of whatever is going on. They have to be able to be truthful. No shame.

Focus group #2, attendee #4

There was general consensus that our planned reimbursement of a US $20 gift card to an online retailer for study surveys three times during the study period was sufficient. However, one participant thought it would be better to provide a gift card to a brick-and-mortar store due to the lack of internet access among the target population.

...people don't have phones, they don't have access to computers, so they can't spend [gift cards] anyway. They just end up selling it on the street for, like, half price or something, you know what I mean? So there's, like, even [names of local grocery and drug stores]...[where] they can use [a gift card]. They’ll get food or whatnot. You know what I’m saying? You can’t really do anything with [name of an online retailer], that’s online.

Interviewee #3

### Using Formative Data to Develop the Protocol and Interventions

The initial version of our study protocol was implemented in June 2018 (see Multimedia Appendix 3). In addition to meeting the criteria for PrEP use at the clinic of enrollment, eligible participants have to be 18 years of age or older; must be MSM or TGP; must be able to understand, read, and speak English; must report methamphetamine use in the past 3 months; must have a cell phone that can send and receive text messages; and must intend to remain in the King County area for at least 6 months. Exclusion criteria include PrEP use in the prior month; circumstances that preclude provision of informed consent, make participation unsafe, or make it unlikely that they would be able to participate for 6 months; or discomfort or anxiety with regard to text messaging. The criterion of discomfort or anxiety with text messaging was a direct result from our formative work.

Study procedures are summarized in Table 2 and the specific intervention components and procedures that resulted from our formative work are listed in Table 3. Briefly, eligible participants who choose to enroll are provided a link to a baseline survey and, if they complete it within 3 days of their initial PrEP appointment, they are randomized to one of the four study interventions: standard of care, text messaging, peer navigation, or combined text messaging and peer navigation. Participants are followed at 1 month, in accordance with the clinic’s procedures; 3 months; and 6 months. Follow-up surveys are sent to participants at 3 and 6 months; for each completed survey, participants are sent a US $20 gift card to an online retailer by email. All participants are invited to participate in an in-depth interview when they complete or discontinue the study. Participants are provided an additional US $40 gift card to an online retailer for completing an in-depth interview. We chose not to include a gift card option to a brick-and-mortar store because we needed the ability to send the gift cards to individuals electronically, since online study surveys are administered remotely and end-of-study interviews can also be done via teleconference or phone.

**Table 2:** Timing of procedures for the Hit Me Up! study.

Procedures and length of clinic visits		Enrollment^a^		Month 1		Month 3		Month 6		Early discontinuation	
**Clinical procedures**											
	HIV testing		X		X		X		X		X
	Hepatitis B surface antigen testing		X								
	Creatinine testing		X		X		X		X		X
	Sexually transmitted infection screening		X				X		X		X
	Adherence counseling		X		X		X		X		X
**Research procedures**											
	Research consent		X								
	Online computer-assisted self-interview		X				X		X		X
	Blood collection for dried blood spots				X		X		X		X
Clinic visit length (min)		90		30		30		30		30	

^a^The research procedures for the enrollment visit may be performed on the same day of the initial pre-exposure prophylaxis (PrEP) clinical visit or on the following day.

**Table 3:** Study procedures implemented based on formative research findings.

Study procedure category	Study procedures
Peer navigation	Require peers to have experience working or networking with substance users, particularly people who use methamphetamine Peer works flexible hours Peer role includes working remotely Peer training includes boundary setting
Text messaging	Study sends three messages per day Various adherence reminders are sent Reminder messages are straightforward Participants select two ranges of 2-hour time periods for messages in baseline survey Participants select content area of some messages in baseline survey Participants select the word they want in their adherence reminders in the baseline survey Text message platform has the ability to send emojis
Educational and recruitment materials	Some cards use “PnP” (party and play) Images used do not overtly show methamphetamine or sex Some materials use symbols indicating inclusion of transgender participants
Other study procedures	Participants with a phobia of technology are excluded Some study clinics have evening and weekend hours

We incorporated suggestions from our formative work into the peer position and training. We included *prior experience working or networking with substance users, particularly methamphetamine users* as a required criterion for the peer position and created a role with flexible work hours and locations. Upon hiring, study onboarding includes standard research training sessions as well as training sessions with specific modules related to peer support, substance use, and boundary setting. Peers also receive ongoing peer support. The peer attempts to meet with all participants randomized to the peer navigation intervention within one week. During this initial contact, the peer completes a harm reduction–based needs assessment (see Multimedia Appendix 4). We anticipated that a peer would be able to provide assistance to 7-10 participants based on a recommendation from leaders of a local peer counselor program for persons who use methamphetamine. However, because the peer navigation is individually tailored and participants have varying levels of need, the study team meets regularly to ensure that the number of participants the peer is assisting is appropriate, and additional peers can be hired as needed.

The peer checks in with participants receiving the peer support intervention at least once per week to help support PrEP persistence, adherence, and study retention. The peer provides tailored support and referrals (eg, to shelters, needle exchanges, etc) in response to participants’ needs, which may include appointment reminders, direct coordination with the pharmacy and PrEP clinic, and providing transportation.

Based on our focus groups and interviews, participants are asked to select the timing of text messages and content areas in the baseline survey. The survey asks participants to select two ranges of 2-hour time periods within 24 hours (eg, 12 AM-2 AM and 2 AM-4 AM) during which to receive the messages. Each participant receives one daily PrEP adherence reminder, one text containing general PrEP information, and a third text from the content areas of their choice. Participants can select as many content areas as they choose, including local social and health services; general health information; harm reduction information for safer methamphetamine use or injection drug use; bondage and discipline, dominance and submission, and sadism and masochism (BDSM) and toys; PnP; STIs; condoms; anal care; and communication. Participants are also asked in the baseline survey which word they prefer for the daily adherence reminder (ie, “PrEP,” “pill,” “medication,” or other); their preference is programmed into their daily text messages.

Since the messages that were reviewed during our formative work aimed to reduce methamphetamine use and sexual behaviors for HIV and were not specific to PrEP, we created additional categories of messages related to PrEP knowledge and adherence. In response to suggestions from the focus groups and interviews, our text messages include emojis and the PrEP reminder texts are straightforward. In addition, we created a variety of PrEP reminder messages so they can vary throughout study participation as recommended by participants in our formative work. Based on the baseline survey selections, text messages are randomly selected from our text library and programmed to be sent at the chosen times. We chose Telerivet [26] as our text messaging platform because it is affordable and has the functionality for two-way messaging, including emojis in text messages, and for uploading personalized text message schedules for participants.

**Figure 1:**
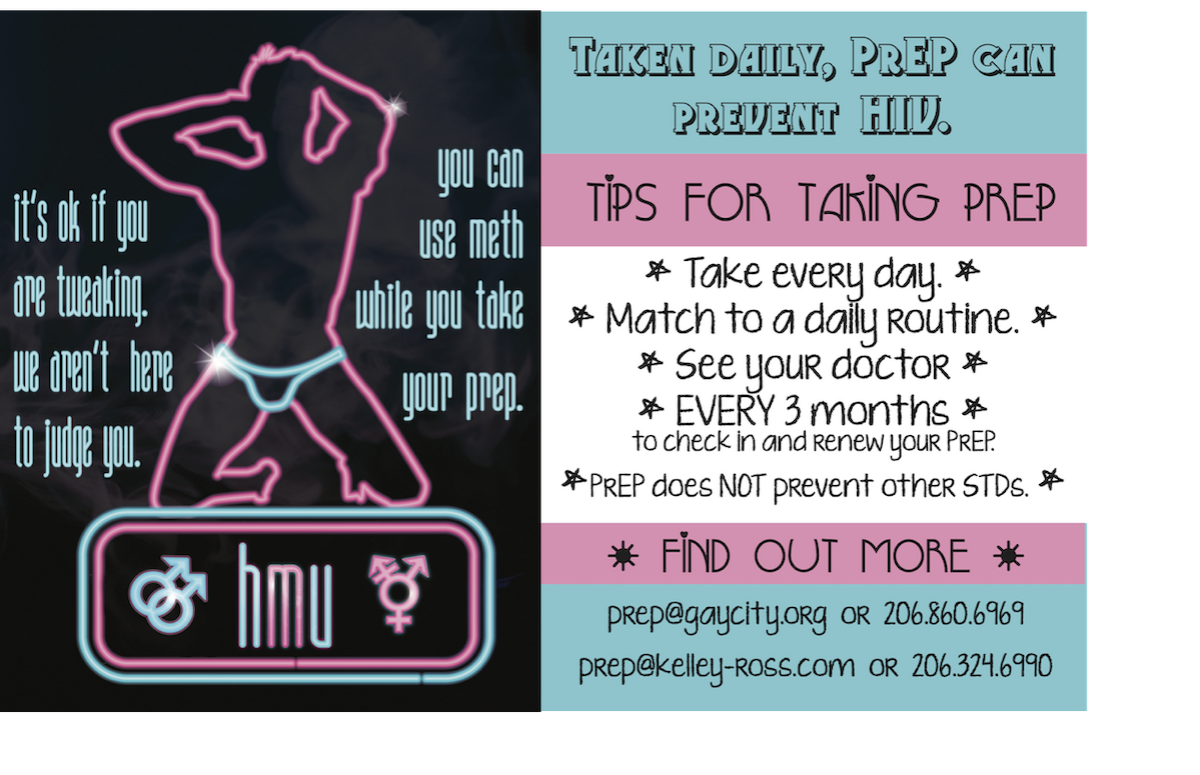
Revised educational cards that include the recommendations of focus group and interview participants. HMU: Hit Me Up; PrEP: pre-exposure prophylaxis; STD: sexually transmitted disease.

Finally, our education and recruitment materials were modified based on the suggestions from focus group and interview participants, as shown in Figure 1.

## Discussion

In this paper, we describe protocol development and implementation for a study that aims to evaluate the acceptability and feasibility of peer navigation and text messaging to support PrEP adherence and persistence among MSM and TGP who use methamphetamine. It is important to involve individuals from the target communities in trial and intervention design in order to optimize participant experience and study outcomes. Prior to fully developing our interventions and study protocol, we conducted focus groups and interviews with the study target populations and then integrated the findings of our formative work into the study interventions and protocol.

Overall, participants thought MSM and TGP would be interested in participating, although they expected recruitment and retention to be challenging due to concerns for stigma, competing priorities, and nontraditional schedules. They shared that the peer navigator should be someone who is nonjudgmental, flexible, and has experience with methamphetamine. There was consensus that three texts a day were appropriate, messages should be straightforward and nonjudgmental, and participants should be able to tailor the timing and content of the messages. We incorporated these recommendations into the peer training process and selection of our text message platform, library, and procedures.

There are limited data regarding peer navigation or intervention development among MSM and TGP who use methamphetamine. Reback et al conducted a formative study to develop text messages specifically for MSM who use methamphetamine to reduce use and high-risk sexual behaviors that were based on behavioral change theories (ie, the Health Belief Model, the Social Support Theory, and the Social Cognitive Theory) [23]. In our study, we assessed the relevance of a subset of those messages with our target study population and found that messages providing informational and emotional support resonated the most with participants. Another study, with young Black men that also used focus groups to refine procedures and content of their HIV-prevention text messaging intervention, found a similar preference for straightforward, factual texts. However, participants in those focus groups recommended including humor throughout the texts, which we did not hear from our participants [27]. A range of text frequencies across different populations have been reported as appropriate; in the study above with young Black men, three messages per week were recommended, whereas another formative study among adolescent MSM found that 8-15 texts per day were acceptable [27,28]. While there was a range in our formative work, three texts per day were within the acceptable range across participants, and more than six would have been unacceptable.

This study has a number of limitations. First, our findings represent the opinions of participants in two focus groups and three interviews, which may not be generalizable to the larger community of MSM and TGP who use methamphetamine in King County, Washington. Moreover, these findings may not be relevant to communities who use methamphetamine outside of Seattle, where PrEP use is prevalent and generally easy to access. Second, our study focused on PrEP adherence and persistence and did not identify barriers to PrEP access. PrEP studies should support MSM and TGP who use methamphetamine across the PrEP cascade. Third, PrEP knowledge and technological advances are changing rapidly over time, and our findings regarding information and technological features that participants desired may be less relevant for interventions being designed today.

Finally, while we tried to include TGP so that we could develop interventions that were culturally appropriate for participants who are not cisgender, we had a difficult time recruiting people for these activities and only had two interviews with individuals who were not cisgender. We also had limited representation of people of color, who are disproportionately impacted by HIV. Future work developing HIV-prevention interventions, including peer navigation and text messaging, should aim to incorporate transgender participants, specifically transgender women and transgender MSM, and persons of color to ensure cultural relevancy to communities at the highest risk for HIV. During the period of this study, we have supported local events for transgender awareness and brought our educational and recruitment materials to in-person spaces and online platforms that may reach more people who are transgender and persons of color, including social media and hookup apps. Our study has a data safety and monitoring board (DSMB), which monitors the study to ensure participant safety. In order to represent needs of study participants who may be especially vulnerable, the DSMB includes TGP of color.

Our study is currently enrolling participants, and results regarding the peer navigation and text messaging interventions to support PrEP use among MSM and TGP who use methamphetamine will be published elsewhere. Through the focus groups and interviews described herein, we aimed to incorporate the opinions and feedback of MSM and TGP who use methamphetamine in the refinement of our interventions and protocol. Incorporating populations most impacted by HIV in the design of PrEP interventions increases their chance for success and thus maximizes PrEP’s potential as a tool for ending the HIV epidemic.

## Appendix

Multimedia Appendix 1 Focus group discussion guide.

Multimedia Appendix 2 In-depth interview discussion guide.

Multimedia Appendix 3 Initial version of study protocol.

Multimedia Appendix 4 Needs assessment.

## Abbreviations

BDSM: bondage and discipline, dominance and submission, and sadism and masochism
DSMB: data safety and monitoring board
HMU!: HIV Prevention in Methamphetamine Users; Hit Me Up!
LGBTQ+: lesbian, gay, bisexual, transgender, and queer or questioning individuals, plus other sexual and gender minorities
MSM: men who have sex with men
NIH: National Institutes of Health
OLE: open label extension
PnP: party and play
PrEP: pre-exposure prophylaxis
Project NEON: Project Needle and Sex Education Outreach Network
SASG: Seattle Area Support Groups
STI: sexually transmitted infection
TGP: transgender people
