# Supplementary material for: Protocol Development for HMU! (HIV Prevention for Methamphetamine Users), a Study of Peer Navigation and Text Messaging to Promote Pre-Exposure Prophylaxis Adherence and Persistence Among People Who Use Methamphetamine: Qualitative Focus Group and Interview Study
Source: JMIR Form Res. 2020 Sep 14;4(9):e18118. doi: 10.2196/18118 (PMC7522731; doi:10.2196/18118)
Supplement: Multimedia Appendix 1 [file formative_v4i9e18118_app1.docx]

**Discussion Guide for Focus Groups for Trial Development**

**Introduction**

Thank you for participating in this activity. We are excited to talk with you about developing a future study that aims to support the use of pre-exposure prophylaxis (PrEP) for HIV prevention among cism and trans* who have sex with men (MGM/TG) and use methamphetamine (meth). The study will enroll people who identify as MSM/TG, use meth, and seek PrEP at Gay City Health Project or Kelley-Ross pharmacy and choose to participate. The study will be voluntary for people who are eligible, and if they do not want to participate they can still receive PrEP at either location outside of the study. The study will evaluate the feasibility and acceptability of a text messaging and peer navigator intervention to help participants take PrEP consistently.

We are hosting this focus group to ask for your input on the messages and materials we are developing for the study, to make sure they are relevant, appropriate, and helpful for TG/MSM who use meth. In addition, we will ask you for any feedback regarding the interventions themselves that may make them more helpful for study participants. We really appreciate you taking the time to talk with us today. This conversation should take approximately 1 hour.

We will audio-record the conversation. We are doing this so that we can listen to the recording and create a written transcript of the conversation. This way we can capture all of the details of the conversation. We ask that during this discussion you try not to use your real name or the real name of anyone else in this room, or the real name of any outreach contacts, to help ensure their confidentiality. In order to help us not use real names, we ask that you write a fake name on the paper that is sitting in front of you and then turn it around so it faces outwards so everyone in the room can see it. This way if someone wants to address you during the conversation, they can use that name. No names will be included on the transcript of this discussion, so if someone’s real name is used during the discussion by mistake, it will not be recorded on the transcript. Once the transcript is completed, we will delete the audio recording. *[The facilitator will pause while the focus group participants write a name on the paper in front of them and place it facing outwards.]*

Please remember, that participating in this focus group discussion is completely voluntary. You may decide to not answer any of the questions that we ask and can leave at any time. Please also remember to be as honest as possible; there are no “right” or “wrong” answers. My job tonight is to help facilitate the discussion. I am eager to hear from everyone and learn from your experiences.

Does anyone have any questions or concerns about the focus group? *[The facilitator will answer any questions that arise.]* Okay, thank you. I am going to start recording the discussion now. *[The facilitator will turn on the audio recorder.]*

*Note: the following questions are a guide for the facilitator. Depending on the conversation, the facilitator may not ask a question, ask questions in a different order, or ask a question that is responsive to the conversation that may not be included in this guide. At all times the facilitator will be responsive to what the participants share and any questions that may arise. The prompts are included to help the facilitator narrow a question or ask it in a different way to help understandability or elicit responses. Additionally, for focus groups that are done with participants who are not representative of the study target population, but have experience working with them (e.g., Project NEON peer educators), the questions will be reframed in the third-person as necessary (e.g., “meth-using MSM/TG” instead of “you”).*

**Questions**

- If you were to participate in the study, what information would you want to be provided?
  - *Prompts:*
    - *Information about PrEP’s safety? Efficacy?*
    - *Information about how to pay for it?*
    - *Information about how to use PrEP correctly?*
- What would be the best materials to provide this educational information? What would they look like?
  - *Prompts:*
    - *Palm cards, flyers, brochures, posters?*
    - *What kind of images should be used? Color scheme?*
- What information about the study would be important to you on recruitment materials? What would make the study interesting to you?
  - *Prompts:*
    - *A description of what research is?*
    - *The role of the peer navigator? Confidentiality?*
    - *How detailed should the description of the study be?*
- What would be the best materials to use for recruitment? What would they look like?
  - *Prompts:*
    - *Palm cards, flyers, brochures, posters?*
    - *What kind of images should be used? Color scheme?*
- Where should these recruitment materials be placed?
  - *Prompts:*
    - *Clinics? Bathhouses? Other venues?*
    - *Online? If so, which websites?*
    - *Handed out in person?*

The study plans to randomly assign participants to one of four interventions: standard of care (what the clinic does anyway, outside of the research study), text messages, peer navigation, or both text messages and peer navigation. All participants will be asked to answer a survey at study entry, 3 months, and 6 months and be reimbursed $20 for each completed survey. *[Facilitator will show following image on handout or will draw on white board if available.]*

| Clinic Standard of Care (SOC) n=10 | SOC + Peer Navigation Intervention n=10 |
| --- | --- |
| SOC + Text Messaging Intervention n=10 | SOC + Combined Intervention n=10 |

- Do you think people would be interested in the study considering that not everyone gets an intervention, but some will only receive standard of care?
  - Prompts:
    - Do you think there is specific messaging we should use to describe these different groups so that participants understand the design? What messages would be important?

Taking PrEP consistently includes going to clinic visits at least every 3 months, taking the pill each day, and refilling your prescriptions monthly or every 3 months. We are interested in determining what messages and activities would be helpful for study participants to do these things. If you were a study participant…

- What would you do to try and remember to take a pill each day?
  - *Prompts:*
    - *Do you have experience with other daily pills? If so, what did you do to help remember taking them? Did you miss pills? If so, what would have helped to not miss them?*
- What kinds of text messages would be helpful to you to take PrEP consistently?
  - *Prompts:*
    - *Reminders to take PrEP?*
    - *Reminders about clinic appointments?*
    - *Reminders about study procedures (e.g., the survey)?*
    - *Reminders and instructions for refilling their PrEP prescription?*
- How many text messages would you want to receive a day? How many would be too many?
- What would you need to remain engaged in PrEP services, including going to the clinic appointments, taking your PrEP pills, and refilling your prescriptions?
  - *Prompts:*
    - *Do you have other experiences where you needed to go to the doctor repeatedly? If so, did anything in particular help you do that?*
    - *Do you have any medications that you need to refill at a pharmacy? If so, what helps remind you to do that?*
- Could a peer navigator help with keeping you engaged in PrEP services? If so, how?
  - *Prompts:*
    - *Accompany you to clinic visits? To the pharmacy?*
    - *Help you with transport?*

*[The focus groups facilitator will then hand out drafts of educational materials and ask participants for feedback about the specific messages.]*

- What do you think of these materials?
  - *Prompts:*
    - *Are they understandable?*
    - *The messages?*
    - *The images?*
- Are these materials relevant to you?
- Would you pick them up?
- Would you call the phone number on them?
- What would you change on these materials?
  - *Prompts:*
    - *Would you use different messages? What would you omit? What would you include?*
    - *Would you use different images? What other images would be relevant and appropriate for you?*
    - *What other information would you include on these materials?*

*[The focus groups facilitator will then hand out draft text messages.]*

- What do you think of these messages?
  - *Prompts:*
    - *Are there certain ones you really like?*
    - *Are there certain ones you really don’t like?*
- Are these messages relevant to you?
- Are there other messages you think would be useful?
  - *Prompts:*
    - *Are there certain topics that would be useful to include in the text messages (e.g., general health, meth use, HIV prevention)?*
- Is there anything else you would like to add regarding the study, the materials, or the messages?

**Thank you very much for sharing your thoughts and opinions!**
