# Supplementary material for: Protocol Development for HMU! (HIV Prevention for Methamphetamine Users), a Study of Peer Navigation and Text Messaging to Promote Pre-Exposure Prophylaxis Adherence and Persistence Among People Who Use Methamphetamine: Qualitative Focus Group and Interview Study
Source: JMIR Form Res. 2020 Sep 14;4(9):e18118. doi: 10.2196/18118 (PMC7522731; doi:10.2196/18118)
Supplement: Multimedia Appendix 3 [file formative_v4i9e18118_app3.docx]

**Interventions to Improve the HIV PrEP Cascade among Methamphetamine Users**

**Study Protocol**

**Version 1.0**

**Sponsored and funded by:**

The National Institute of Drug Abuse (NIDA)

**Principal investigator:**

Joanne Stekler, MD, MPH

Department of Medicine

University of Washington, Seattle, WA

**Table of Contents**

[1. Introduction 4](#_Toc512144065)

[2. Sites 4](#_Toc512144066)

[3. Eligibility 4](#_Toc512144067)

[3.1 PrEP Eligibility Criteria 4](#_Toc512144068)

[3.2 Additional Study Inclusion Criteria 5](#_Toc512144069)

[3.3 Exclusion Criteria 5](#_Toc512144070)

[4. Visit Procedures 5](#_Toc512144071)

[4.1 Screening, Informed Consent, and Release of Information 5](#_Toc512144072)

[4.1.1 Screening 5](#_Toc512144073)

[4.1.2 Informed Consent and Release of Information 5](#_Toc512144074)

[4.2 Entry Visit Procedures 6](#_Toc512144075)

[4.3 Randomization 6](#_Toc512144076)

[4.4 Study Follow-up 7](#_Toc512144077)

[4.5 Table 1. Study Procedures 8](#_Toc512144078)

[4.6 Early Discontinuation 8](#_Toc512144079)

[5. Interventions 8](#_Toc512144080)

[5.1 Text Messaging Intervention 8](#_Toc512144081)

[5.2 Table 2. Description of Text Message Categories 9](#_Toc512144082)

[5.3 Peer Navigation Intervention 9](#_Toc512144083)

[6. Data sources 10](#_Toc512144084)

[6.1 Text Messaging Intervention 10](#_Toc512144085)

[6.2 Peer Navigation Intervention 10](#_Toc512144086)

[6.3 Online CASI Surveys 10](#_Toc512144087)

[6.5 Clinical Data 11](#_Toc512144088)

[6.6 DBS Testing 11](#_Toc512144089)

[6.7 Cost-effectiveness 11](#_Toc512144090)

[6.8 Participant Interviews 11](#_Toc512144091)

[7. Adverse Events (AEs) 11](#_Toc512144092)

[7.1 Definition of an AE and SAE 11](#_Toc512144093)

[8. Data Management 12](#_Toc512144094)

[8.1 Data Management 12](#_Toc512144095)

[8.2 Quality Assurance 12](#_Toc512144096)

[8.3 Data Security 13](#_Toc512144097)

[9. Human Subjects Considerations 13](#_Toc512144098)

[9.1 Institutional Review Board (IRB) 13](#_Toc512144099)

[9.2 Staff Training 13](#_Toc512144100)

[9.3 Consent 13](#_Toc512144101)

[9.4 Study Materials and Messages 13](#_Toc512144102)

[9.5 Potential Risks and Methods to Reduce Risks 14](#_Toc512144103)

[9.5.1 HIV testing 14](#_Toc512144104)

[9.5.2 Finger sticks 14](#_Toc512144105)

[9.5.3 Sensitive Information 14](#_Toc512144106)

[9.5.4 Loss of confidentiality 14](#_Toc512144107)

[9.6 Benefits 15](#_Toc512144108)

[9.7 Data and Safety Monitoring Board (DSMB) 15](#_Toc512144109)

# Introduction

Like the HIV care continuum, the PrEP continuum spans multiple sequential steps, from entry of HIV-negative persons into the continuum by HIV testing; to being aware, initiating, and adhering to PrEP; and leading finally to the prevention of HIV acquisition. Local methamphetamine (meth)-using men and trans who have sex with men (MSM/TG) are at very high risk of HIV acquisition and, based on our preliminary data, appear to have high awareness of PrEP (97%) and high rate of insurance coverage (97%). Yet few meth-using MSM/TG seek initial PrEP prescriptions, and even fewer persist. This study will evaluate the acceptability and feasibility of a text messaging and peer navigation intervention to support PrEP initiation, daily adherence, and persistence over a six-month study period among MSM/TG who seek PrEP at one of two clinics in Seattle.

**Specific aims:**

Aim 1. Conduct a pilot randomized control trial (RCT) to evaluate the acceptability and feasibility of a peer navigation intervention for meth-using MSM/TG

Aim 2. Evaluate acceptability and impact of two-way text messaging on meth use and PrEP adherence.

# Sites

The study will include two clinical sites where participants will be enrolled and have their follow-up visits. These two sites are the Gay City Wellness Center and Kelley-Ross Pharmacy. The Principal Investigator leads a weekly PrEP clinic at Gay City and Kelley-Ross operates a pharmacy-based PrEP program called One-Step PrEP. The administrative site will be the University of Washington (UW) offices located at the Ninth and Jefferson Building. The study will store dried blood spot (DBS) specimens at the UW Retrovirology Laboratory. Appendix A lists each site’s location and contact information.

# Eligibility

This study will enroll eligible and interested participants starting PrEP at either Gay City or the Kelley-Ross pharmacy. First, eligible participants must meet the study site’s PrEP eligibility criteria, and then they must meet the additional study eligibility criteria. Participants will be screened for eligibility by staff at the study site, and staff will complete an eligibility checklist to ensure all participants enrolled are eligible and that the eligibility assessment is recorded.

## PrEP Eligibility Criteria

- Gay City:
  - Report at least one of the following:
    - Sex without condoms in a serodiscordant relationship
    - In last year:
      - Condomless anal sex with HIV+ man
      - Condomless anal sex with man of unknown HIV status
      - Diagnosis of gonorrhea, chlamydia, or syphilis
      - Used meth
      - 10 or more oral or anal male sex partners
- Kelley-Ross:
  - Report at least one of the following:
    - Any anal sex without condoms (receptive or insertive) in past 6 months
    - Any STI diagnosed or reported in the past 6 months
    - Is in an ongoing sexual relationship with an HIV-positive male partner
    - Entering dating scene and anticipating having condomless anal sex

## Additional Study Inclusion Criteria

- 18 years of age or older
- HIV-negative
- Cisgender man or individual on the trans gender variant spectrum who has sex with men
- Ability to understand, read, and speak English
- Reports meth use in the past 3 months
- Has a cell phone able to send and receive text messages
- Intends to remain in the area for at least 6 months

## Exclusion Criteria

Individuals who are screened and meet ANY of the following criteria will not be eligible for study enrollment:

- PrEP use in the prior month
- Discomfort or anxiety with regards to text messaging
- At study entry has any circumstances that, based on the opinion of the clinical staff, would preclude provision of informed consent, make participation unsafe, or make it unlikely the participant would be able to participate for 6 months

# Visit Procedures

## Screening, Informed Consent, and Release of Information

### Screening

People who meet the PrEP eligibility criteria, identify as MSM/TG, and report using meth in the past 3 months will be asked if they would like to participate in the study. For those who demonstrate interest, the site staff will assess their study eligibility following the inclusion and exclusion criteria detailed in section 3. The eligibility assessment will be recorded on an eligibility checklist. The eligibility checklist can be found in Appendix B with other study forms.

If a person is interested and deemed eligible, the site staff will provide them a consent form, enroll them in the study, and complete the entry visit procedures. The only research procedure conducted at the clinic during the entry visit is informed consent, the other procedures are for the clinical care of the participant and are done in accordance with the site’s operating procedures.

### Informed Consent and Release of Information

Informed consent is a process in which an individual voluntarily expresses their willingness to participate in research, and to undergo the procedures after having been informed of all of the aspects to the research that are relevant to their decision. The requirements for informed consent in federally funded research, like this study, can be found in 45 Code of Federal Regulations (CFR) 46 and 21 CFR 50. Informed consent must precede any research procedures and the documentation of consent and the date it was obtained is critical.

In order to obtain consent, the clinical staff will meet with an interested and eligible participant in a private setting and review the Institutional Review Board (IRB)-approved consent form with them. They will ensure that the participant knows that participating in research is voluntary and that they can stop their participation at any time. Once the consent form is reviewed with the participant, the clinical staff will only obtain the participant’s signature if they are confident that all aspects of the consent are clearly understood by the client. Once the consent is reviewed, all questions have been answered, and the staff is confident the client understands the procedures, the client and the staff will both sign and date the consent form.

In addition to the study consent, participants will need to complete a HIPAA Authorization and release of information (ROI). The ROI will be completed between the sites and UW so that the research team can access the participants’ clinical records. The ROI should detail that all records and laboratory results relevant to the individual’s PrEP use at the clinic can be shared with the UW research team. The HIPAA Authorization and ROI must be signed at the entry visit.

## Entry Visit Procedures

After the consent form and ROI are signed, the site staff will perform the remainder of the clinical and research entry visit procedures. The visit procedures for the entry visit are shown in Table 1. The entry visit is estimated to take approximately 90 minutes to complete.

The clinical procedures, including a blood draw for laboratory testing, STI screening, and adherence counseling will be performed in accordance with the clinic’s standard of care. At the entry visit, laboratory testing will include creatinine, HIV, and hepatitis B surface antigen (HBsAg) testing. In addition, blood will be collected via fingerstick for dried blood spots (DBS) as described in Section 6.6 for research purposes.

Once a participant has completed their entry visit, the site personnel will fax a copy of the completed eligibility checklist; signed consent, HIPAA Authorization, and ROI; and participant’s contact information to the study team located at UW. The site staff will alert the UW research team via email that a person has been enrolled and the entry visit information has been faxed over.

One of the research coordinators will obtain the faxed documents and create patient folders for the participant. One folder will include all of the materials that have identifying information (e.g., signature page of consent, ROI, and contact information) and the other will be for storing de-identified study materials. The folder with identifying information will be stored in a locked file cabinet in Dr. Stekler’s office and the other one will be stored in a locked file cabinet at the research coordinators’ desk.

Then one of the research coordinators will enter the new participant into the study text platform along with their contact information and send the participant a unique link to the baseline survey.

## Randomization

Once the participant has completed the baseline survey they will be randomized. The research coordinator will open a sequentially numbered, sealed envelope with the study allocation (generated by blocked randomization with variable block sizes and random number generator). They will record the treatment assignment of the participant in their de-identified chart and send a text to inform the participant of their assignment. All participants entered into the text platform will be put into groups based on their random assignment. *It is important that they are placed into their treatment group in the database at the point of randomization to prevent any intervention spillover.*

In accordance with our 2x2 study design (Figure 1) we will randomly assign 10 participants to each arm: 1) standard of care, 2) text messaging only, 3) peer navigation only, and 4) text messaging and peer navigation.

Figure 1. 2x2 Factorial Design

| Clinic Standard of Care (SOC)  n=10 | SOC +  Peer Navigation Intervention  n=10 |
| --- | --- |
| SOC + Text Messaging Intervention  n=10 | SOC + Combined Intervention  n=10 |

## Study Follow-up

Follow-up visits will be scheduled from the participant’s entry visit, at 1 month, 3 months, and 6 months. The procedures to be performed at follow-up visits are shown in Table 1. The only research procedures conducted during a participant’s follow up are the CASI, blood collection for DBS, and those related to their assigned intervention arm if they are randomized to text messaging or peer navigation. For participants in the standard of care arm, there are no additional research procedures aside from the CASI and blood collection at the sites. All other procedures are done in accordance with the sites’ clinical operating procedures as part of standard PrEP follow-up. At all visits, participants will receive HIV and creatinine testing as well as adherence counseling as part of their clinical follow-up. In addition, at months 3 and 6 participants will receive STI screening as standard of care. Blood will be collected for research purposes using a fingerstick to collect DBS samples at all follow-up visits. Participants will also be asked to complete an online CASI survey at months 3 and 6. We anticipate each follow-up visit to take approximately 30 minutes and online CASI to take 20 minutes.

Reminder calls will be done by the study site staff as part of their regular PrEP follow-up. At Gay City, clients receive a visit reminder the day before and the day of the visit. At Kelley-Ross, clients receive a reminder email one week and one day before the visit. If a client misses an appointment, the clinical staff will contact them to try to reschedule their appointment as soon as possible.

## Table 1. Study Procedures

## Early Discontinuation

While the study team will do their best to retain all participants for the duration of the study, it is possible that participants will discontinue their participation early, before the final 6-month visit. This could occur due to participant choice, study team decision, or experiencing an adverse event (AE). If someone has an AE related to PrEP and is willing to remain in the study, we will discontinue PrEP in that participant but continue to follow them to monitor the AE and its resolution. If someone exits the study early, we will attempt to schedule a final visit that will include the same procedures as a scheduled month 3 or 6 visit. If a participant refuses to return for a final visit, we will ask them to at least complete an exit CASI. Reasons for early discontinuation will be recorded on study data collection forms.

In addition, the study team will discontinue participants if they do not complete a baseline CASI within 3 days of their entry visit, in which case they will be replaced. All participants who end the study early will be invited to participate in a qualitative interview (Section 6.8)

# Interventions

The two interventions that are being assessed in the study are text messaging and peer navigation. We will randomly assign 10 enrolled participants to each of these interventions separately, and another 10 to a combined intervention of both. These interventions are described briefly below, with additional information regarding data collection and extraction in the Data Source section of the protocol (Section 6). Furthermore, more operational details can be found in the Procedural Manual.

## Text Messaging Intervention

Participants who are assigned to receive text messaging (n=20) will receive three texts per day from the study platform. The first text will be sent within 24 hours of randomization. The study will use a two-way messaging platform that allows participants to reply to messages that are received. While the text messaging only intervention does not encourage two-way messaging, we want participants to be able to communicate with study staff as needed, and we will follow-up with any received texts as appropriate.

Participants will be asked to choose a time of day that they prefer to receive texts, and we will send texts during their selected time. The texts will be selected from a text library. Participants will receive one daily text that will concern PrEP adherence and then can choose which categories they would like for the other two messages. These categories are described in Table 2. If a participant leaves the study early, the research coordinator will block any remaining texts being sent to that individual at the time of their discontinuation.

If a participant assigned to the text messaging intervention either does not have the ability to continue receiving texts during the study period or loses their phone during the study and cannot replace it, the study will cover the costs associated with continued phone and text access for the participant.

## Table 2. Description of Text Message Categories

## Peer Navigation Intervention

Participants assigned to the peer navigation intervention (n=20) will receive support from the peer navigator. These activities include, but are not limited to, in-person meetings, appointment reminders and escort, refill assistance, and check-ins about adherence. The peer will introduce themselves to participants in this intervention arm within 24 hours of randomization via their preferred method of contact (as recorded at the clinic during the entry visit). The peer will schedule their first in-person meeting within 1 week of enrollment.

As some meth users will have greater functioning than others, the peer will create an individualized plan for each subject and tailor activities and resources based on acuity. Individualized plans will be completed at study enrollment and exit to be able to record needs and progress during the study. Additional individualized plans may be completed during follow-up at the discretion of the peer and depending on the needs and acuity of the participant.

# Data sources

## Text Messaging Intervention

The text messaging platform provides data export files in multiple formats that include the person the messages is to/from, content of message, date and time, whether the message is scheduled or not, and other technical information. We will use these data exports to track the text messages that are sent and received throughout an individual’s participation.

## Peer Navigation Intervention

Data from the peer navigation intervention will be collected through multiple methods as described below:

- The text platform data exports will document the texts between the peer navigator and participant.
- The individual plans that the peer completes throughout each participant’s participation will be a record of the participant’s acuity and ongoing needs assessment. A copy of the individualized plan form can be found in Appendix B.
- In addition all peer navigation activities that are not recorded on the text platform or via the individual plans will be recorded in a log that the peer will complete, including additional texts, phone conversations, and face-to-face contacts. A copy of the peer interaction log can be found in Appendix B.

## Online CASI Surveys

Online CASI surveys will be administered via REDCap. All participants will receive them at baseline, month 3, and month 6. Surveys will collect information on participants demographics, HIV risk factors, substance use, depression, self-efficacy, PrEP adherence, experiences with stigma, social support, provider relationship, and intervention acceptability. All participants will receive a $20 Amazon gift card for each completed survey. Data is readily exportable from REDCap in a variety of formats with associated codebooks.

- 1. Adherence Assessments

PrEP adherence will be assessed primarily by DBS, but we will also collect self-reported adherence at the clinic by staff and via the online CASI. At Gay City all participants are asked the number of missed pills in the past 7 days and 30 days at their follow-up visits. At Kelley-Ross participants are asked their number of remaining pills and the number of pills missed in the past month and 3 months.

## Clinical Data

Any data obtained as a part of clinical standard of care procedures will be collected and maintained in accordance with study site policies and procedures. We will extract data regarding PrEP appointments and refills from the clinical charts. We will ensure that once we extract information from the clinical chart the data is de-identified and coded with the participant’s study ID.

## DBS Testing

Blood for DBS will be collected at months 1, 3 and 6 and if someone leaves the study early. DBS will be collected via a fingerstick in accordance with the Colorado Antiviral Pharmacology Laboratory DBS by Finger-stick Collection SOP. All staff will wear disposable gloves and use universal safety precautions when they collect fingerstick specimens and process DBS. The DBS cards will be labeled with the participant’s ID, date of collection, and protocol name and will be air-dried for at least 2 hours and up to overnight at the clinic. Dried cards will be placed in a bag with a humidity indicator card and desiccant pack to reduce humidity. A study team member will obtain the DBS bags from the clinical sites and bring them to UW where they will be stored in the UW Center for AIDS Research Clinical and Retrovirology Research Core until the end of the study. DBS will be batch shipped once all participants have completed their participation to the Colorado Antiviral Pharmacology Laboratory for drug level testing.

## Cost-effectiveness

We will collect data to perform cost-effective analyses of the study interventions. Cost-effectiveness data will be collected across a variety of sources, including training logs, service costs, logs of peer interactions with participants, time and motion studies of staff, and interviews with the study team. The research coordinator will track expenses for the project, including those needed for cost-effective analyses and log time required for site trainings. The peer will be responsible for recording their training and logging the time and costs associated with any peer navigation activities. Approximately 6 months after study start the study team will collaborate with a UW investigator to conduct interviews with the peer and time and motion studies at the sites.

## Participant Interviews

The research coordinator will conduct semi-structured interviews with all willing study participants after they finish study follow-up to better understand the acceptability and feasibility of the study interventions and participants experience with PrEP. Interviews will be digitally audio-recorded and transcribed verbatim, and identifying information will be deleted. Qualitative analysis of transcripts will begin with a conventional content analysis. Participants who discontinue the study early and choose not to participate in an interview will be asked for the reason of discontinuation, which will be recorded.

# Adverse Events (AEs)

## Definition of an AE and SAE

An adverse event (AE) is defined as “any unfavorable, unintended diagnosis, symptom, sign (including an abnormal laboratory finding), syndrome, or disease that occurs during the study, having been absent at baseline, or—if present at baseline—appears to worsen.” (Interim Guidelines for NIH Intramural Principal Investigators and for NIH Institutional Review Boards on Reporting Adverse Events.) AEs can be non-medical, including legal, housing, or employment problems; substance use; and violence. Both clinic staff and the peer navigator will assess for AEs and may identify them during a participant’s participation.

Any AE that meets **one** of the following criteria is considered a serious adverse event (SAE):

- results in death, or
- is life-threatening (places the patient at risk of death), or
- requires hospitalization or prolongs an existing hospitalization, or
- causes persistent or significant disability or incapacity, or
- requires medical intervention to prevent one of the above outcomes.
  1. Reporting AEs and SAEs

Though we do not expect any such occurrences, any AEs, SAEs, or unanticipated problems will be reported to the principal investigator immediately. If the peer navigator or any other study staff is informed of any such events before clinic staff, then clinic staff should also be notified immediately so that clinic care can be adjusted accordingly. AEs will be reported to the NIDA program officer (PO) as part of the annual progress report. SAEs will be reported by email to the NIDA PO within 24 hours of the event, including a brief explanation of the SAE. A detailed follow up, submitted within 72 hours of the event, will include the following information:

- date of the event
- what occurred
- actions taken by project staff
- planned follow up (if any)
- the intervention group/study arm of the affected participant
- whether the event appears to be related to the intervention
- whether the participant will continue in the study

Reporting policies for the UW Human Subjects Division and IRBs can be found here: https://www.washington.edu/research/hsd/study-activities/report-events-and-new-information/guide-to-reporting-new-information/

# Data Management

## Data Management

The data will be managed by the study coordinator in cooperation with clinicians. Data will be protected by the use of a study ID to protect identifiable patient information. Each participant will be assigned a sequential study ID upon enrollment. The link between their identifiable information and study ID will be stored in a secure office at UW. The sources of data are described in Section 6.

## Quality Assurance

The research coordinator will monitor for adequate subject recruitment and enrollment. The data manager will monitor the validity/integrity of data. Data that is collected at all study visits will be reviewed by the data manager for completeness within a week of each visit. Signed research consents, HIPAA Authorizations, and ROIs will be collected from study sites and reviewed for completion. If data were not collected or there is missing data, the data manager will follow-up as appropriate, which may include contacting the study sites or one of the study staff to resolve the problem. The research coordinator will remind participants to fill out 3 and 6 month CASIs if they have not been completed. Any other, non-urgent, issues that arise will be discussed at the regular monthly meetings with study team and PI. The research coordinator and data manager will cooperate to perform bi-weekly checks on data collected to ensure quality and security.

## Data Security

Access to the file cabinet where identifiable data are stored will be provided only to study staff whose functions require this access (the peer navigator, research coordinator, and principal investigator). Electronic databases with de-identified data including survey information will be stored in a secure, password protected digital format that will be developed and maintained by the data manager in conjunction with all study staff. Online CASIs will be hosted on a secure, HIPAA compliant website.

The phone numbers of participants who are assigned to the peer navigation intervention will be stored on the peer’s encrypted and password-protected phone. However, the logs and individual plans that the peer completes will be de-identified and only include participants’ study IDs.

# Human Subjects Considerations

## Institutional Review Board (IRB)

Human subjects will be enrolled in this study and approvals from the University of Washington IRB will be obtained prior to initiating any study procedures. The requirements and procedures of the UW IRB can be found on the website for the UW Human Subjects Division (HSD) at: <https://www.washington.edu/research/hsd/>.

This study will not include the involvement of special vulnerable populations (fetuses, neonates, pregnant women, children, prisoners, and/or institutionalized individuals).

## Staff Training

Any study staff who perform research procedures will have completed human subjects training.

## Consent

As described in Section 4.1.2, informed consent will be obtained prior to study entry. The informed consent form will be approved by the IRB and the stamped copy will be used during the enrollment visit. Study staff will review the consent form with eligible and interested participants and answer any questions that they have. Prior to obtaining consent the staff member will assess the potential participant’s understanding of the study procedures, risks, and benefits and will only consent individuals who demonstrate understanding of the study. All consent forms will be signed and dated by both the participant and staff member.

## Study Materials and Messages

The study team will attempt to develop and disseminate study materials and messages that are inclusive, respectful, and relevant to MSM/TG individuals who use meth. Prior to study initiation, the study team performed formative research with members of the MSM/TG community who use meth and stakeholders to review materials and messages to increase their usefulness, inclusiveness, and relevance to the target community.

## Potential Risks and Methods to Reduce Risks

### HIV testing

The most significant potential risk in this study is that a participant could test positive for HIV. Participants are tested for HIV at their PrEP visits as a part of routine standard of care. A positive HIV test could be a significant psychological stressor for a patient. In this event, the participant would receive standard clinical care, counseling, support, and referrals to help them cope with the diagnosis. Clinic staff are experienced and well-trained professionals who are well versed in HIV testing and counseling. Clinic staff and the study team are also familiar with local resources and will connect participants with additional resources as needed.

### Finger sticks

The fingerstick for blood collection may cause a small amount of pain or bruising, and the site may bleed slightly after collection. The study staff will follow standard procedures for collecting blood specimens from participants and attempt to minimize any chance for discomfort or bruising.

### Sensitive Information

The CASI will ask questions about drug use and sexual practices. Participants could experience some discomfort while taking the survey, due to the sensitive nature of some of the questions. Participants will be encouraged to find a private space they can take the surveys. We will send a unique link for each survey to each participant so that we can track who completes the surveys without asking participants to enter any identifying information into the survey.

### Loss of confidentiality

The study staff will do everything possible to keep participants’ information private; however, there is always the risk of a breach of confidentiality. All identified study documents will be kept in a locked cabinet in the principal investigator’s locked office. De-identified study records will be stored in a locked cabinet at the research coordinators’ desk and on work computers that are password-protected and encrypted. The link between the study ID and identifying information will be stored in a secure office at UW. Participants’ phone numbers will be stored in the secure text platform to send links to study surveys, and text messages for those assigned to the text message intervention.

All members of the research team are responsible for the security and protection of research records. Procedures of preventing breaches of confidentiality include regular Collaborative Institutional Training Initiative (CITI) training of all study staff, collecting minimum necessary identifiers, limiting access to identifiers, encrypting devices, and using coded data. If there is a breach of confidentiality, we will report this to the IRB as an unanticipated problem within 24 hours and will address the breach to a degree that is dependent on the extent and impact of the breach. Consequences may include staff retraining, changing of staff responsibilities, up to dismissal of study staff for serious violations.

## Benefits

Participants who are assigned to either receive text messaging, peer navigation, or both will have the benefits associated with these interventions. We anticipate that these groups will all have better adherence to PrEP, which is associated with better PrEP efficacy and HIV prevention. Participants will also receive support and referrals to additional resources around reducing their meth use if they are interested. The participants who are assigned to the standard of care will not receive any direct benefit from research participation in this study.

## Data and Safety Monitoring Board (DSMB)

The study will have a DSMB to ensure the safety of individuals participating in this project, since the study will enroll a population (MSM/TG who use meth) that is considered vulnerable. The DSMB will review the protocol prior to the start of the trial and meet annually until follow-up has been completed. During the trial, they will review all adverse events, complaints, and study outcomes to ensure that the interventions are not associated with increased harms. In case of an SAE, the DSMB members will be sent a notification of the event within 24 hours and asked to decide independently whether an emergent meeting and review is necessary in response to the SAE.
