# Supplementary material for: Protocol Development for HMU! (HIV Prevention for Methamphetamine Users), a Study of Peer Navigation and Text Messaging to Promote Pre-Exposure Prophylaxis Adherence and Persistence Among People Who Use Methamphetamine: Qualitative Focus Group and Interview Study
Source: JMIR Form Res. 2020 Sep 14;4(9):e18118. doi: 10.2196/18118 (PMC7522731; doi:10.2196/18118)
Supplement: Multimedia Appendix 4 [file formative_v4i9e18118_app4.docx]

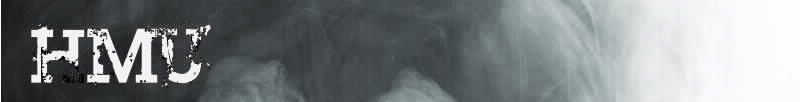
 **Participant Status Sheet**

**Study ID #_______________ Date _____________________ Visit # _______________**

**Instructions**: For each area ask participant about where they are at right now and also where they would like to be at.

| Area | Current Status | Desired Status |
| --- | --- | --- |
| **Basic Needs** |  |  |
| **Food** |  |  |
| **Shelter** |  |  |
| **Risk Awareness (HIV)** |  |  |
| **Transportation** |  |  |
| **Health** |  |  |
| **Self Sufficiency** |  |  |
| **Mental Health** |  |  |
| **Harm Reduction (Drugs, Alcohol)** |  |  |
| **Sexual Health** |  |  |
